# Activities at the Universal Protein Resource (UniProt)

**DOI:** 10.1093/nar/gkt1140

**Published:** 2013-11-16

**Authors:** The UniProt Consortium

**Affiliations:** ^1^European Molecular Biology Laboratory, European Bioinformatics Institute (EMBL-EBI), Wellcome Trust Genome Campus, Hinxton, Cambridge CB10 1SD, UK, ^2^SIB Swiss Institute of Bioinformatics, Centre Medical Universitaire, 1 rue Michel Servet, 1211 Geneva 4, Switzerland, ^3^Protein Information Resource, Georgetown University Medical Center, 3300 Whitehaven Street North West, Suite 1200, Washington, DC 20007, USA and ^4^Protein Information Resource, University of Delaware, 15 Innovation Way, Suite 205, Newark, DE 19711, USA

## Abstract

The mission of the Universal Protein Resource (UniProt) (http://www.uniprot.org) is to provide the scientific community with a comprehensive, high-quality and freely accessible resource of protein sequences and functional annotation. It integrates, interprets and standardizes data from literature and numerous resources to achieve the most comprehensive catalog possible of protein information. The central activities are the biocuration of the UniProt Knowledgebase and the dissemination of these data through our Web site and web services. UniProt is produced by the UniProt Consortium, which consists of groups from the European Bioinformatics Institute (EBI), the SIB Swiss Institute of Bioinformatics (SIB) and the Protein Information Resource (PIR). UniProt is updated and distributed every 4 weeks and can be accessed online for searches or downloads.

## INTRODUCTION

UniProt’s mission is to facilitate scientific discovery by organizing biological knowledge and enabling researchers to rapidly comprehend complex areas of biology. The four UniProt databases are optimized for different users as follows: the UniProt Knowledgebase (UniProtKB) is an expertly curated database consisting of two sections: a reviewed section containing manually annotated records with information extracted from literature and curator-evaluated computational analysis (UniProtKB/Swiss-Prot), and an unreviewed section with automatically annotated records (UniProtKB/TrEMBL); the UniProt Archive (UniParc) (1) is a comprehensive sequence repository, reflecting the history of all protein sequences not only in the UniProtKB but also in all the source databases; the UniProt Reference Clusters (UniRef) (2), which merge closely related sequences based on sequence identity to facilitate sequence similarity searches; and the UniProt Metagenomic and Environmental Sequence (UniMES) database, which was created to cater for the developing area of metagenomics. This article describes UniProt’s biocuration and how we are responding to the ever increasing amount and complexity of the data being generated in the genomic and proteomic era and ensuring that we continue to deliver excellence to the scientific community.

## NEW AND ONGOING DEVELOPMENTS

### UniProt biocuration

**Figure 1.**
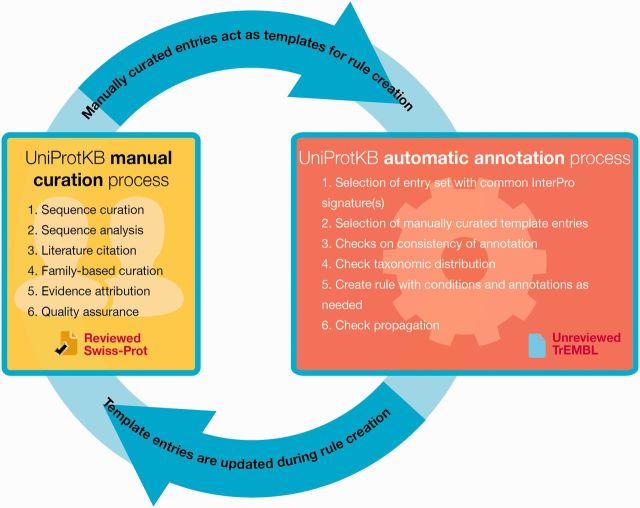
UniProtKB manual and automatic biocuration processes.

*Manual and automatic annotation in UniProtKB*.**UniProt leads the world in providing full and comprehensive curation of the experimental data in the literature and does this in a mutually beneficial collaboration with other specialized resources. UniProt curation is not only added to UniProtKB but is actively provided for leverage and integration into other resources through their Web sites and curation pipelines. For example, UniProt’s protein nomenclature is used in NCBI’s Reference Sequence collection (RefSeq) (3) and the INSDC (ENA/DDBJ/GenBank) (4) submission guidelines. This highly coordinated approach to curation means that effort is not duplicated. Literature-based expert curation of UniProtKB provides high-quality information for experimentally characterized proteins in a standardized and structured way using widely accepted controlled vocabularies and ontologies. UniProt concentrates its manual annotation effort on annotating experimental data from the literature for reference proteome records and aims to provide high-quality annotation for representative members of all protein families across diverse taxonomic groups. Reference proteomes cover the proteomes of well-studied model organisms and other proteomes of interest for biomedical research (http://www.uniprot.org/taxonomy/complete-proteomes), which ensures we address the needs of a diverse user community and maximize the inclusion of data with the highest scientific impact. Records are curated following the process shown in Figure 1. For more detailed descriptions of our processes and priorities, see http://www.uniprot.org/help/biocuration and http://www.uniprot.org/docs/sop_manual_curation.pdf. The goal of a fully manually curated UniProtKB record is to describe all known protein products for a given gene for a given species (+strain) and to provide a summary of published experimental characterization for each protein product. Data captured from the literature include protein and gene names, function, catalytic activity, cofactors, pathway information, subcellular location, protein–protein interactions, pattern of expression and phenotypes or diseases associated with variants in a protein. Curation of the protein sequence includes functional sites or regions, as well as variant protein forms produced by natural genetic variation, RNA editing, alternative splicing, proteolytic processing and post-translational modifications (PTMs).

UniProt has built a team of biocurators and developers to enable the optimum approach to automatic annotation. We have developed two complementary systems to automatically annotate uncharacterized protein sequences with a high degree of accuracy. The first system, UniRule, consists of manually curated annotation rules. UniRule provides a consistent framework for incorporating annotation rules created by the Consortium members. Biocurators who carry out the curation of experimentally characterized proteins also create and maintain rules for automatic annotation of uncharacterized proteins, and the developers ensure that the supporting technical infrastructure, including the UniRule curation tool, enables these rules to be applied both accurately and efficiently and are kept fully up-to-date with each UniProt release. UniRule is complemented by the Statistical Automatic Annotation System (SAAS), a completely automatic decision-trees-based rule-generating system. Rules from both systems specify annotations as well as the conditions that must be satisfied for them to be applied. The InterPro hierarchy (5) of protein family and domain signatures is used as the basis for protein classification and along with other conditions such as taxonomy and sequence length, triggers application. Two of the UniProt partners create signatures [the HAMAP (6) and PIRSF (7) resources] for integration into InterPro for the UniRule system, which greatly enhances its efficiency. Together these systems currently predict protein properties such as nomenclature, function, catalytic activity, pathways, subcellular location and sequence-specific information such as the location of ligand-binding and active sites, for approximately one-third of UniProtKB/TrEMBL. Annotation from the automatic annotation systems is labeled with evidence attribution indicating both the system and the specific source rule on both the UniProt Web site and in the XML format of UniProtKB available at http://www.uniprot.org/downloads.

*Gene ontology annotation project.* In 2013, the UniProt-GO annotation dataset has grown with the inclusion of five new sources of manual annotation. These annotations are to proteins from *Aspergillus* by the AspGD project (6), from the *Pseudomonas aeruginosa* Community Annotation Project (PseudoCAP) (9), from archaeal and bacterial species by the Microbial Energy processes Gene Ontology Project (MENGO http://www.mengo.bioinformatics.vt.edu), from multiple species by the Community Assessment of Community Annotation with Ontologies project (CACAO) (10) and human and mouse transcription factors by the Systems Biology team at the Norwegian University of Science and Technology (NTNU). We have also started producing all of our species-specific and multispecies annotation files in the new GO Consortium Gene Product Association Data (GPAD) and Gene Product Information (GPI) formats. This is of particular benefit to users of the UniProt multispecies annotation file, as the new formats allow us to provide considerably smaller files compared with the Gene Association File (GAF2.0) format. All annotation files are available from our ftp site at ftp://ftp.ebi.ac.uk/pub/database/GO/goa. Additionally, we now release a set of specific annotation files that are based on a version of a subset of the UniProt Reference proteomes that provide one protein product per gene, therefore reducing the redundancy of annotations to multiple UniProtKB records for a single gene identifier. Our curation interface Protein2GO has been recognized by the Gene Ontology Consortium (GOC) as an extremely powerful tool that has many inbuilt quality control checks and is easy to use. As such, some members of the GOC [WormBase (11) and *Saccharyomyces* Genome Database (SGD) (12)] have started to use Protein2GO as their primary GO curation tool, and we expect more groups to join in the near future. All of this annotation is added to UniProtKB. With regard to our manual curation, we embarked on a project to curate, using GO, the biological roles of 88 human peroxisomal proteins. This was driven by the establishment of a link between peroxisomal metabolism and human disease. This project illustrates the usefulness of manually curated annotations and how these can help organelle-specific biological data analysis as well as facilitating cross-species comparisons with similarly curated datasets (13). We also had a successful collaboration with members of the Systems Biology team at NTNU, Mouse Genome Informatics (MGI) and SGD to draw up published guidelines for GO annotation of mammalian transcription factors (14).

### Developments in UniRef

The UniProt Reference Clusters (UniRef) provide sets of sequences from UniProtKB (including isoforms) to obtain complete coverage of the sequence space at several resolutions while hiding redundant sequences (but not their descriptions) from view. In 2013, the intra-cluster coherency was improved by introducing an 80% overlap threshold to the computation of UniRef90 and UniRef50 clusters. The threshold ensures that the longest (seed) sequence of each UniRef90 and UniRef50 cluster has a minimum length overlap of 80% with each of the other members. The overlap prevents intra-cluster incoherencies such as the ones observed in clusters with polyproteins and the homologs of individual proteins with various molecular functions. The overlap threshold is not used in UniRef100 to continue removing sequence redundancy resulting from fragments identical to full-length proteins. The change had a small impact on cluster topologies in UniRef50 (<5% increase in number of clusters and <2% of representatives change) while leading to more than 5-fold reduction in UniRef50 computation time. In addition to the cluster improvements, a new full and incremental update calendar was adopted. Going forward, full computation of clusters will be done once a year at the beginning of the year, and there will be incremental updates throughout the year.

### Growth of UniProt–challenge of how to respond to the complete genome era

**Figure 2.**
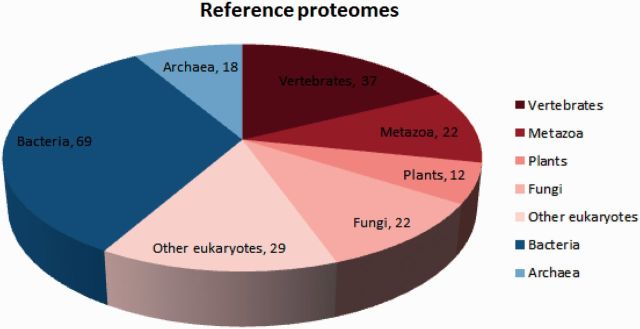
Reference proteomes (excluding viruses) in UniProtKB.

UniProt continues to rapidly grow with release 2013_10 containing 45 288 084 entries. As described in an earlier article (15), UniProt has responded to the increasing submission of multiple versions of sequences from similar organisms and strains by developing the concept of complete and reference proteomes. This is to ensure that users will only find the most relevant and best-annotated protein sequences when searching instead of drowning in reports of redundant sequences. A complete proteome is defined as the entire set of proteins expressed by a specific organism. The majority of the UniProt complete proteome sets are based on translations of completely sequenced genomes, and will normally include sequences that derive from extra-chromosomal elements such as plasmids or organellar genomes in organisms where these occur. UniProt has selected a subset of these complete proteomes to be references. Reference proteomes provide a broad coverage of the tree of life, and we intend for them to constitute a representative cross-section of the taxonomic diversity to be found within UniProtKB. They include the proteomes of well-studied model organisms and other proteomes of interest for biomedical and biotechnological research. Species of particular importance may be represented by numerous reference proteomes for specific ecotypes or strains of interest. The UniProt genomic pipeline uses INSDC, Ensembl (16) and RefSeq as sources for the underlying complete genomes. UniProt works closely in collaboration with our colleagues in those groups to map all the UniProtKB proteins to the underlying genomic assemblies and to define together the same complete and reference genomes/proteomes. By September 2013, we have defined 3686 complete proteomes and 595 reference proteomes in UniProtKB (see Figure 2). We are going to speed up the designation of reference proteomes by using UniRef to provide computationally selected reference proteomes. We also plan to develop pan-proteomes to capture unique sequences not found in a taxonomic group’s reference proteomes. Within a taxonomic group, there are often other non-reference proteomes containing unique sequences not found in the group’s reference proteome. This will address the requirement for a representative set of all the sequences in a taxonomic group from users interested in proteome diversity, gene evolution, gene transfer and phylogenetic comparisons. As mentioned earlier, we will be providing versions of the reference proteome datasets with one representative protein (usually the best annotated) per gene for download, which will address the needs of various users such as the Model Organism Databases who annotate to the gene. The amount of redundancy continues to increase with the increasing release of closely related genomes, particularly for bacteria but also for many viruses and eukaryotes. To deal with this flow, we will not add highly redundant proteomes (e.g. in UniProt release 2013_07, there were 1 386 943 sequences submitted for strains of *Escherichia coli and Enterococcus faecalis)* to UniProtKB but make them available instead through UniParc. We will extend UniParc to include annotation data, which will allow the user to retrieve meaningful datasets for these proteomes. We will provide proteome identifiers that will uniquely identify a protein set for the assembly of a completely sequenced genome.

### Variation in UniProtKB

As described earlier, the UniProt genomic pipeline enables the linking from all UniProtKB records processed by this pipeline to the genomic resources at Ensembl, INSDC and RefSeq. Linking UniProtKB to the genomic sequence provides new opportunities to explore the wealth of genomics, to characterize a diversity of species genomic landscapes and to identify natural variants and mutations relevant for certain diseases. For example, UniProt has annotated the complete *Homo sapiens* proteome and ∼20 000 protein-coding genes are represented by a canonical protein sequence in UniProtKB/Swiss-Prot, with some records describing alternative isoform sequences. Most of these protein sequences have now been mapped to the reference genome assembly that is produced by the international Genome Reference Consortium through Ensembl cross-references. This has allowed the genomic pipeline to identify single nucleotide polymorphisms from the 1000 Genomes human data stored in the Ensembl *H. sapiens* variation database that describe a variation that alters the encoded protein sequence with resulting biological consequences in some cases. We plan to extend the pipeline to other species for which Ensembl maintains variation databases. These are built by harvesting data either from Single Nucleotide Polymorphism Database, COSMIC (Catalogue of Somatic Mutations in Cancer) (17) and UniProtKB or by using files provided by collaborators. As well as developing this import pipeline, UniProt curators annotate information on human variants that are associated with disease states directly from the literature, linking each positional variant feature annotation to the relevant description from a controlled vocabulary of almost 4000 human diseases (http://www.uniprot.org/diseases). Information on polymorphisms and our vocabulary of human diseases are also made available in the form of separate files for download (http://www.uniprot.org/docs/humsavar and http://www.uniprot.org/docs/humdisease). Disease descriptions are provided with links to other resources such as the Online Mendelian Inheritance in Man (OMIM) database (18) as well as the Medical Subject Heading (MeSH) descriptors. UniProtKB/Swiss-Prot currently includes ∼24 000 curated variants linked to >3000 proteins involved in disease.

### Proteomics in UniProtKB

Data from high-throughput proteomics experiments constitute a rich potential source of annotations for UniProtKB, providing supporting evidence for the existence of specific protein isoforms and PTMs. These data are made available to the scientific community through the ProteomeXchange Consortium, which includes the PRIDE Proteomics Identifications Database (19). ProteomeXchange provides standard submission and dissemination pipelines for different types of proteomics data (including both tandem MS and SRM data), and a number of standard formats for data exchange. Within ProteomeXchange, PRIDE acts as the initial data submission point and repository for primary data submitted by authors. We have developed a pipeline that can produce UniProtKB annotations using proteomics datasets sourced from repositories such as PRIDE (or other members of the ProteomeXchange consortium) in commonly used formats such as mzTab (20). Published datasets are evaluated for biological relevance and quality by expert curators at UniProt and relevant metadata—including minimal information on sample treatment, instrument acquisition settings and data analysis parameters—is recorded. Peptide identification data are then extracted and experimental peptides that uniquely match the product of a single gene are identified by comparison with a database of all possible theoretical peptides within the target UniProtKB proteome. High-quality unique peptides are then used to generate UniProtKB annotations describing PTMs and protein processing events, and to confirm protein existence. These annotations remain linked to their source peptide(s) and can be easily removed as selection criteria evolve, a feature which will lessen the impact of false-positive identifications (which may accumulate when many individual datasets are combined) (21). We have used our pipeline to completely re-annotate existing protein modifications in UniProtKB using data from >60 published manuscripts describing large-scale experiments in *Homo sapiens*, *Mus musculus*, *Rattus norvegicus* and *Saccharomyces cerevisiae*. In total, >91 000 distinct unique experimental peptides were used to produce 29 600 individual PTM annotations in >9700 individual UniProtKB/Swiss-Prot records. Future work will focus on the incorporation of additional quality controls and metrics from PRIDE such as peptide-spectrum match reliability scores derived from global (cross-platform) clustering of experimental spectra (22). Post processing of the original data in this way is expected to further reduce the incidence of erroneous annotation due to false-positive peptide-spectrum matches, and will help guarantee the continued quality of annotations derived from high-throughput proteomics experiments in UniProtKB. In tandem with these developments, we also plan to make proteomics-based annotation from our pipeline available in UniProtKB/TrEMBL records.

### Highlighting the UniProt Web site

We have been working on redesigning the UniProt Web site following a user-centered design process, consulting >150 users worldwide with different backgrounds and research interests. User-centered design is an iterative approach, which takes into account the requirements and expectations of users at every stage. The redesign consisted of four main stages. We started by analyzing the user experience of the current UniProt Web site to identify issues and gaps. This was followed by the design phase where we created different versions of possible solutions. We prototyped the designs and tested them with users, iterating based on user feedback throughout the testing phase. Changes were then implemented according to the designs and the new site was further evaluated with users. We aim to have this beta site linked from http://www.uniprot.org in the first quarter of 2014. We found that the main issues that users encountered were with the usability and visibility of some existing functionality. We focussed on creating better navigation, improving the visibility of functionality and changing some terminology to be more user-friendly. To make it easier for users to traverse UniProt data, we created easily accessible filters on all results pages and a more effective advanced search query builder. Another area of improvement was the UniProtKB protein entry view, which is the most used page on the Web site. We observed users struggling to navigate long entries to find the information they were looking for. Current section headings like ‘Protein properties’ and ‘General comments’ are based on the flat file format, and our research showed their meanings were not obvious to users. We designed a new entry structure with the content presented under biological headings such as ‘Function’, ‘Interaction’, ‘Subcellular location’. We evaluated this with users and implemented the changes on our new site. We complemented this change with a more usable navigation bar to help users to navigate the page. We also improved the workflow of our tools, e.g. ‘Retrieve’ and ‘ID Mapping’ have been combined under one tool called ‘Upload lists’. This provides one unified entry point for users coming to UniProt with multiple identifiers. Other work on tools included creating a more graphical representation of the Blast output. We are already gathering user feedback on this beta site to make any necessary improvements before it becomes the master site. To get prompt access to the beta site, users should subscribe to the UniProt twitter feed (@uniprot), where we announce our latest news and updates.

### Cross-references and ID mapping

UniProtKB contains extensive cross-references to other databases including sequence databases, model organism databases, pathway databases and various ‘omics’ resources. This addition of a broad spectrum of cross-references ensures that UniProtKB acts as a central hub for biomolecular information by connecting to other resources, which provide additional or complementary information. Cross-references are integrated in close and ongoing collaboration with the particular resources and updated on a regular basis by automatic procedures and supported by expert curation where necessary. UniProtKB has >140 cross-references to other resources. Some notable additions in the last year are GeneWiki (23) and the Protein Ontology (24). Extensive cross-references allow UniProt to provide a service to map different types of identifiers (IDs) across resources that have unique IDs. This service currently supports mappings for >90 unique ID types and enables mapping from non-UniProt IDs to UniProt IDs (and vice versa) and has proved to be a very popular resource (both on the Web site and as a download). As new resources with unique IDs are added as cross-references, they are also added to the mapping service. Some ID mappings, notably NCBI gi numbers, are maintained without explicit cross-references in the UniProtKB records.

### Format changes

Owing to the growth of data in UniProtKB, we will need to extend the accession number format in 2014. The new format will be announced soon in http://www.uniprot.org/docs/sp_soon.htm.

## DATABASE ACCESS AND FEEDBACK

The http://www.uniprot.org Web site (25) is the primary access point to our data and documentation and offers tools such as full text and field-based text search, sequence similarity search, multiple sequence alignment, batch retrieval and database identifier mapping. The home page features a site tour as a quick introduction for novice users. The full text search allows quick and easy searching without prior knowledge of our data or search syntax. The results are sorted by relevance and search suggestions are provided, where possible, to help filter searches that yield too many or no results. More complex queries can be built with the field-based text search, either iteratively with a query builder or by entering them manually in the query field, which can be faster and more powerful (http://www.uniprot.org/help/text-search). Searching with ontology terms is assisted by auto-completion, and search results can be browsed by ontologies. The display of the result sets, as well as database entries, is configurable: columns can be added to or removed from the result table to see more functional annotation than is available in the default display. Sequence similarity search results can be filtered by taxonomy to obtain a quick overview of the taxonomic distribution of the results and the sequence annotations of the matched entries can be projected onto the sequence alignments to see at a glance if important positions are conserved. The site has a simple and consistent URL scheme that allows the bookmarking of all searches to repeat them at a later time. All result sets can be downloaded to offer users the possibility to retrieve customized datasets. However, large downloads are given low priority to ensure that they do not interfere with interactive queries, and they can therefore be slow compared with downloads from the UniProt FTP server. Therefore, we recommend downloading complete datasets from ftp.uniprot.org/pub/databases/uniprot/. The Web site offers various download formats (e.g. plain text, XML, RDF, FASTA, GFF), which depend on the chosen dataset. The tab-delimited and Excel formats can be customized by selecting the desired columns in the graphical view of the result table. All data are also available in RDF (http://www.w3.org/RDF/), a W3C standard for publishing data on the Semantic Web. Both data and search results can also be accessed programmatically, either via simple HTTP (REST) requests (http://www.uniprot.org/faq/28) or via our Java API (UniProtJAPI) (26).

Although the UniProt Web site provides a query interface for all UniProt data, some users also require facilities to search across related data in different databases. Therefore, we have set up a BioMart (http://www.biomart.org) instance at http://www.ebi.ac.uk/uniprot/biomart/martview that allows complex queries between UniProt and other data resources such as PRIDE, Ensembl and InterPro. The UniProt Protein DAS Server provides access to sequence and annotation from UniProtKB and UniParc at http://www.ebi.ac.uk/uniprot-das. To offer users even more flexibility, we are going to provide a SPARQL (http://www.w3.org/TR/rdf-sparql-query/) endpoint for all our data that can be linked with any remote data resource that has a SPARQL endpoint, using SPARQL 1.1’s federated query capabilities. This new service is available for beta testing at beta.sparql.uniprot.org/.

Your feedback is extremely valuable to help us improve our databases and services in terms of accuracy and usability. If you have questions or suggestions, please contact us via http://www.uniprot.org/contact or email us directly at help@uniprot.org. You can also follow us on facebook and twitter (http://www.facebook.com/uniprot.org and @uniprot). You can submit new data or updates at http://www.uniprot.org/help/submissions. Extensive documentation on how to best use our resource is available at http://www.uniprot.org/help/. UniProt is freely available for both commercial and non-commercial use. Please see http://www.uniprot.org/help/license for details. New releases are published every 4 weeks except for UniMES, which is updated only when the underlying source data are updated. Release statistics are available at http://www.uniprot.org.

## FUNDING

The National Institutes of Health (NIH) [UniProt 4U41HG006104-04; GO 5U41HG002273-13; 5R01GM080646-08, 5G08LM010720-03 and 5P20GM103446-13]; British Heart Foundation [SP/07/007/23671]; Swiss Federal Government through the State Secretariat for Education, Research and Innovation; EC [GEN2PHEN (200754) and MICROME (222886)]; National Science Foundation (NSF) [DBI-1062520] and EMBL core funds. Funding for open access charge: National Institutes of Health [4U41HG006104-4].

*Conflict of interest statement*. None declared.
